# Differences in peritoneal response after exposure to low-GDP bicarbonate/lactate-buffered dialysis solution compared to conventional dialysis solution in a uremic mouse model

**DOI:** 10.1007/s11255-018-1872-3

**Published:** 2018-05-04

**Authors:** M. Vila Cuenca, E. D. Keuning, W. Talhout, N. J. Paauw, F. J. van Ittersum, P. M. ter Wee, R. H. J. Beelen, M. G. Vervloet, E. Ferrantelli

**Affiliations:** 1 Department of Nephrology, VU University Medical Center, Amsterdam, The Netherlands; 2 Amsterdam Cardiovascular Sciences, Amsterdam, The Netherlands; 3 Department of Molecular Cell Biology and Immunology, VU University Medical Center, Amsterdam, The Netherlands

**Keywords:** Peritoneal dialysis, Macrophage, IL17, Fibrosis, Angiogenesis

## Abstract

**Background:**

Long-term exposure of conventional peritoneal dialysis (PD) fluid is associated with structural membrane alterations and technique failure. Previously, it has been shown that infiltrating IL-17-secreting CD4+T cells and pro-fibrotic M2 macrophages play a critical role in the PD-induced pathogenesis. Although more biocompatible PD solutions are recognized to better preserve the peritoneal membrane integrity, the impact of these fluids on the composition of the peritoneal cell infiltrate is unknown.

**Materials and methods:**

In a uremic PD mouse model, we compared the effects of daily instillation of standard lactate (LS) or bicarbonate/lactate-buffered solutions (BLS) and respective controls on peritoneal fibrosis, vascularisation, and inflammation.

**Results:**

Daily exposure of LS fluid during a period of 8 weeks resulted in a peritoneal increase of αSMA and collagen accompanied with new vessel formation compared to the BLS group. Effluent from LS-treated mouse showed a higher percentage of CD4^+^ IL-17^+^ cell population while BLS exposure resulted in an increased macrophage population. Significantly enhanced inflammatory cytokines such as TGFβ1, TNFα, INFγ, and MIP-1β were detected in the effluent of BLS-exposed mice when compared to other groups. Further, immunohistochemistry of macrophage subset infiltrates in the BLS group confirmed a higher ratio of pro-inflammatory M1 macrophages over the pro-fibrotic M2 subset compared to LS.

**Conclusion:**

Development of the peritoneal fibrosis and angiogenesis was prevented in the BLS-exposed mice, which may underlie its improved biocompatibility. Peritoneal recruitment of M1 macrophages and lower number of CD4^+^ IL-17^+^ cells might explain the peritoneal integrity preservation observed in BLS-exposed mouse.

## Introduction

Continuous and long-term treatment with peritoneal dialysis (PD) promotes an inflammatory response which eventually leads to a progressive remodeling of the peritoneal membrane [1]. These changes are characterized by the accumulation of extracellular matrix, angiogenesis, and other structural alterations of the peritoneum resulting in technique failure and serious clinical complications including encapsulation peritoneal sclerosis [1]. To a large extent, these events seem to be driven by high glucose degradation products (GDPs) content, the low pH, and the presence of lactate, typically present in conventional PD fluids [2]. Therefore, the conversion of PD fluids towards more biocompatible solutions is recognized as an urgent unmet clinical need to better preserve the peritoneal integrity. As a consequence, lactate–bicarbonate buffered fluids with more physiological pH, a lower amount of GDPs, and alternative solutions in which glucose is replaced by alternative osmotic agents such as icodextrin and amino-acids have been developed [3].

The introduction of neutral pH and particularly bicarbonate/lactate-buffered solutions seemed to offer advantages in terms of peritoneal membrane preservation and peritoneal homeostasis control [4, 5]. In the Euro balance trial, it demonstrated a significant improvement in effluent markers of peritoneal membrane integrity, a decrease in systemic advanced glycation end products (AGEs) levels, less decline in residual renal function and a decrease in peritoneal ultrafiltration [6]. Moreover, such biocompatible fluids increase mesothelial cell markers, induce less systemic inflammation, and reduced the incidence of peritonitis [7–10].

Recent studies revealed that immunological responses underlie PD-induced peritoneal injury upon conventional high GDPs lactate PD fluid exposure although the specific mechanism remains unclear. These data pointed to the importance of IL-17-mediated inflammation as a novel player in the PD-induced injury in both PD patients and experimental models [11]. Furthermore, M2 macrophages are suggested to play a key role in the development of peritoneal inflammation and fibrosis [12, 13]. However, while experimental and clinical data suggest a better preservation of peritoneal morphologic and functional features upon bicarbonate/lactate solution compared to conventional PD solutions, the implication on the inflammatory cell population in this novel solution has been poorly defined. To overcome this knowledge gap, our recently developed uremic mouse PD exposure model [14] was used in the present study to compare a pH neutral low-GDP bicarbonate/lactate-buffered solution (BLS) with a standard high GDPs lactate (LS) PD fluid in respect of inflammation, fibrosis, and vascularisation. Here, we demonstrate that the enhanced fibrotic and angiogenic response observed in LS-exposed mouse was prevented upon BLS exposure. This preservation of the peritoneal integrity by BLS was accompanied with a lower number of CD4^+^ IL-17^+^ cells, higher levels of macrophage-related pro-inflammatory cytokines and with a higher ratio of M1 macrophages over M2 subset.

## Methods

### Mouse PD model

C57BL/6J female mouse (Charles River, Maastricht, The Netherlands) aged 12–14 weeks and weighing approximately 20 g at the start of the study were used. Animals were organized as follows: 1 healthy control group (*n* = 10), 3 PD groups (*n* = 10 per group) daily exposed to 2 ml saline or standard lactate-buffered solution (Dianeal®, Baxter) or bicarbonate/lactate-buffered solution (Physioneal®, Baxter) during a period of 8 weeks. Mouse in all the PD groups underwent 5/6 nephrectomy and catheter implantation (Customized mouse catheter MMP-4S-061108A, Access Technologies, Ridgeway, USA). *5*/6 nephrectomy consisted in the complete removal of the right kidney and the removal of the anterior and posterior 1/3 part of the left kidney by using a monopolar electric blade as previously described [14]. All the animals were housed under standard conditions and were given food and water ad libitum. Health conditions were checked daily. The experimental protocols were approved by the Animal Welfare Committee at the VU University Medical Center, Amsterdam.

### Cell counting

At the end point, following the injection of 2-ml standard PD fluid via a catheter, peritoneal effluents were collected after 30 min; cells were isolated by centrifugation and counted and stained with fluorochrome-conjugated mouse-specific antibodies against CD3, CD4, CD8α, B220, CD11b, Ly6C, F480, and IL-17 purchased from eBiosciences. Before intracellular staining, cells were re-stimulated for 4 h with 50 ng/ml Phorbol 12-Myristate 13-Acetate (PMA) and 500 ng/ml ionomycin in the presence of 1 µg/ml BD Golgi Plug (eBiosciences). Samples were analyzed in a BD FACS Fortessa (BD Biosciences) flow cytometer and further analyses were performed with FloJo software.

### Histology and immunohistochemistry

Parietal peritoneal biopsies were collected from the opposite side from the catheter installation. The biopsies were fixed in Bouin’s solution, embedded in paraffin, cut into 5-µm sections and stained with Masson’s Trichrome. Peritoneal membrane thickness was determined using a Carl Zeiss Microscope (GmbH, 37081, Göttingem, Germany). Microscope photographs were obtained by using an AxioCam ICc5. The peritoneal thickness of each animal was calculated by the median of measurement taken every 50 µm from one side to the other of the tissue sample.

Biopsies were frozen in Tissue-Teck® (O.C.T.® Sakura) and cut into 5-µm sections. To identify myofibroblasts and vessels, samples were stained for anti-rat Alpha Smooth Muscle Actin (αSMA 1A4, DAKO, 1:500) combined with anti-mouse-IgG (H+L) (Invitrogen) and Cluster of Differentiation 31 (αCD31, PECAM, Serotec, Oxford, UK, 1:1000) coupled to anti-mouse-IgG-555 according to the manufacturer’s instructions. Nuclei were stained with DAPI. Fluorescence microscopy was performed with a Carl Zeiss Microscope and photographs were taken with an AxioCam HR R3. The areas positive for CD31 were calculated by CellProfiler software (2.1.1, Broad Institute, UK).

### Immunoblotting

Lysates of the peritoneal membrane were prepared by homogenizing of preserved tissue in lysis buffer containing Protease Inhibitor Cocktail (Roche Applied Science, Indianapolis, IN). Protein concentrations were determined using the Pierce Micro BCA Protein Assay Kit (Thermo Scientific, Rockford, IL). The following antibodies were used: CD31 (Abbiotec; 1: 200), Collagen I (Abcam; 1:250), GAPDH (14C10) 1:1000 cell signaling, followed by donkey anti-rat/rabbit conjugated with HRP (Dako; 1:5000). Signal was visualized using enhanced chemiluminescence (Life Sciences) on LAS3000 (Fujifilm, Japan). Image J (NIH, Bethesda, Maryland) was used for analysis.

### Quantification of cytokines

Peritoneal effluents collected after 8 weeks of treatment were made cell free by centrifugation (300G, 5 min, RT) and stored at − 20 °C. Protein levels of mouse Transforming Growth Factor β 1 (TGFβ1), Interleukin-1β (IL-1β), Tumor Necrosis Factor α (TNFα), Vascular Endothelial Growth Factor (VEGF), IL-17A, IL-6, Interferon γ (INFγ), IL-5, IL-4, Macrophages Inflammatory Proteins 1α and β (MIP-1α and MIP-1β) were quantified by ProcartaPlex™ Multiplex Immunoassays (Affymetrix eBioscence).

### Statistical analysis

Data were analyzed using GraphPad Prism software (La Jolla, CA). Statistical analysis was performed using One-way ANOVA test to compare the groups. A *P* value < 0.05 was considered statistically significant (*P < 0.05, **P < 0.01, ***P < 0.001). Data were shown as means ± SD.

## Results

### BLS exposure in mouse prevented the development of both peritoneal fibrosis and angiogenesis with no changes in thickness

**Fig. 1:**
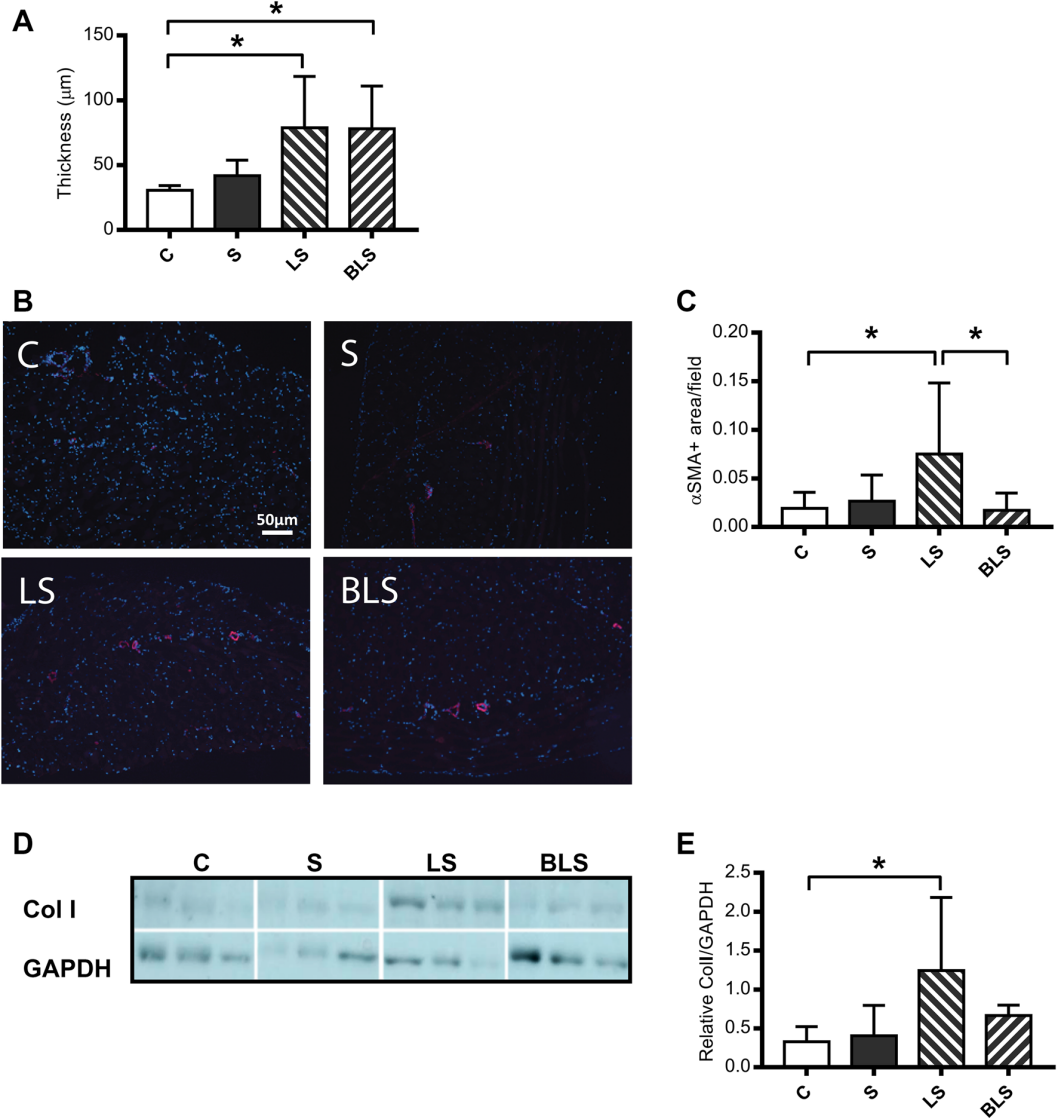
Exposure to low GDPs bicarbonate/lactate-buffered PD fluid prevents myofibroblast recruitment but does not protect from parietal peritoneum thickness. Graph **a** represents peritoneal thickness (µm) measurements for each group (*n* = 10). Representative immunofluorescence microscopy (**b**) and analysis (**c**) of parietal peritoneal sections stained with αSMA marker (magnification ×10; *n* = 10). αSMA positive cells are indicated in red. Nuclei were stained with DAPI (blue). Representative immunoblotting (**d**) and analysis (**e**) of Collagen I (Col I) levels in total protein lysates of peritoneal membrane. GAPDH was used as loading control (*n* = 6). *C* control, *S* saline, *LS* lactate PD fluid, *BLS* bicarbonate/lactate PD fluid. Data show means ± SD. Differences were considered statistically significant for *P* < 0.05 using one-way ANOVA. *P < 0.05

In order to mimic the situation in chronic kidney disease patients undergoing PD, a uremic mouse model, performed by 5/6 nephrectomy, was exposed to daily PD fluid during a period of 8 weeks. Nephrectomized groups showed a twofold increase in both serum urea and creatinine concentrations 15 days after the surgery and remained stable for the duration of the experiment (data not shown). As shown in Fig. 1a, after 8 weeks of daily exposure to the PDFs, there was a statistically significant increase (*P* = 0.04) in peritoneal thickness compared to the non-PDF-exposed control (C: 30.63 ± 3.66, S: 41.84 ± 12.05, LS: 78.78 ± 39.62, BLS: 78.11 ± 32.87). However, thickening of peritoneum did not differ between the two PDF compositions (LS or BLS). In contrast, immunohistological analysis of peritoneal biopsies revealed that exposure to BLS significantly prevented (*P* = 0.01) the accumulation of α αSMA positive cells (myofibroblast) in the parietal membrane when compared to conventional LS PDF (C: 0.020 ± 0.016, S: 0.026 ± 0.026, LS: 0.075 ± 0.072, BLS: 0.016 ± 0.014), (Fig. 1b, c). Similarly, statistically significant enhanced collagen I protein levels were detected in peritoneal samples after exposure to LS fluid when compared to control, whereas upon BLS no significant effect was shown (C: 0.33 ± 0.19, S: 0.40 ± 0.39, LS: 1.24 ± 0.94, BLS: 0.66 ± 0.13), (Fig. 1d, e). Analysis of the peritoneal effluents collected after 8 weeks of daily exposure to the different treatments performed by standard peritoneal equilibrium test (PET) revealed no differences between the groups regarding the volume of ultrafiltration (data not shown).

**Fig. 2:**
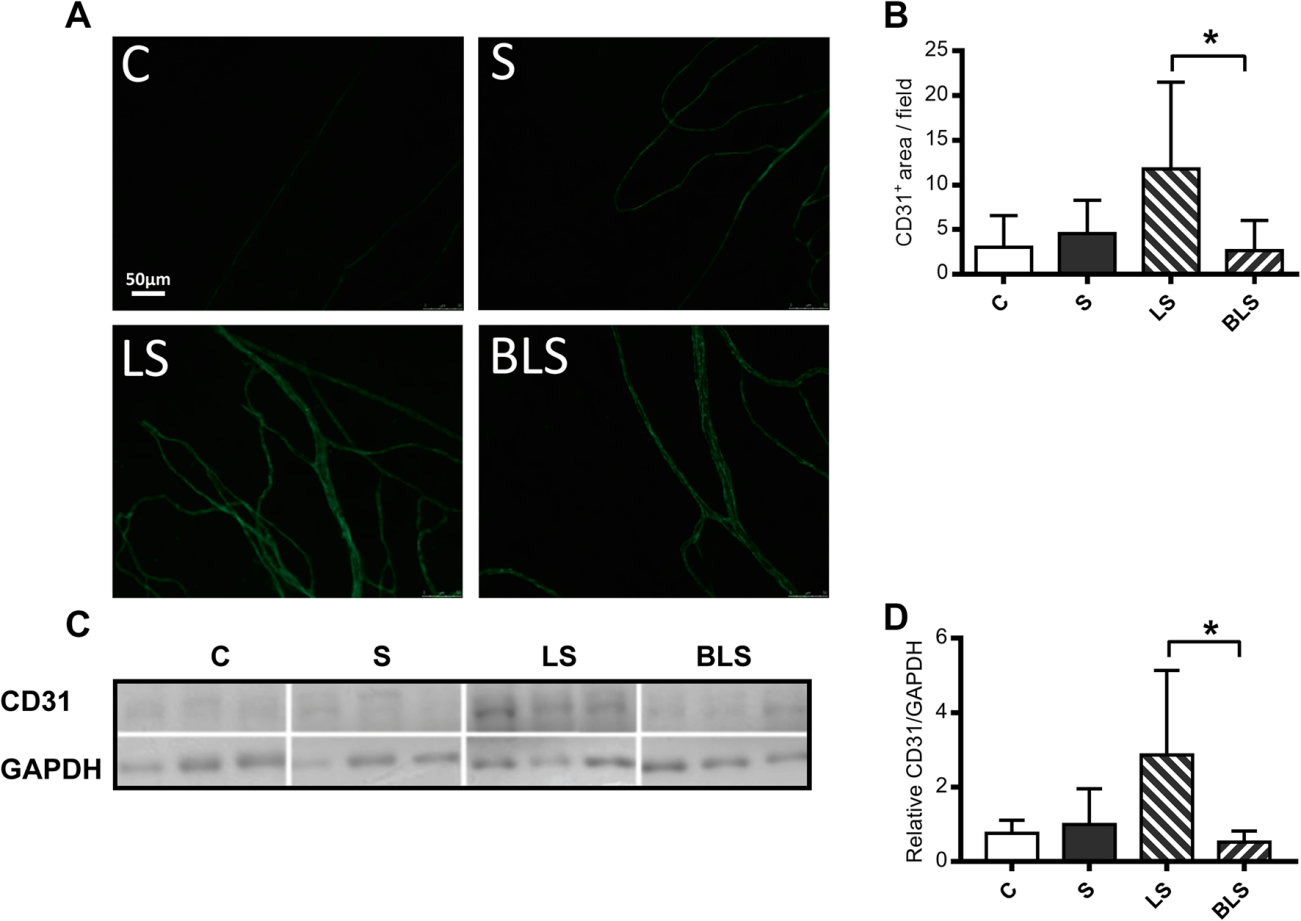
Peritoneal angiogenesis is attenuated in bicarbonate/lactate-buffered-exposed mouse. Immunofluorescence microscopy (**a**) and analysis (**b**) of omentum sections stained for vasculature with CD31; (green) marker (*n* = 10). Each value corresponds to an average (% surface staining CD31) of 10 independent values of each mouse omentum taken each time (magnification ×20). Representative immunoblotting (**c**) and analysis (**d**) of CD31 levels in total protein lysates of peritoneal membrane (*n* = 6). GAPDH was used as loading control. *C* control, *S* saline, *LS* lactate PD fluid, *BLS* bicarbonate/lactate PD fluid. Data show means ± SD. Differences were considered statistically significant for *P* < 0.05 using one-way ANOVA. *P < 0.05

Chronic treatment with LS resulted in a significant increment (*P* = 0.02) of new vessel formation in the omentum, represented by the increased abundance of CD31 protein-positive cells (indicative of endothelial cells) when compared with the BLS group (Fig. 2a, b). These data were confirmed by the detection in the peritoneal tissue of high levels of CD31 protein upon LS fluid which was prevented (*P* = 0.02) in BLS-exposed animals (Fig. 2c, d). Taken together, these results indicate that LS but not BLS fluid promoted a fibrotic and angiogenic response in the peritoneum although the development of increased peritoneal thickness was not preserved in BLS-exposed mouse.

### Analysis of inflammatory cell recruitment in peritoneal cavity upon PD fluid exposure

Exposure to PD fluid caused a numerical increase of the total number of cells in the peritoneal fluid compared to non-exposed animals, which however was not statistically significant (Table 1). No statistically significant variation was found in both CD4- and CD8-positive populations among groups while there was a ninefold statistically significant increase in CD4/IL-17 double positive lymphocytes (CD4^+^ IL-17^+^) found in the LS group compared to the BLS group, in which these double positive cells remained at the same level as controls. No significant differences were observed between the groups in the percentage of B cells and monocytes (Ly6G^+^ CD11b^+^), although the number of the latest increased especially after BLS. As in our previous study in mouse [15], a slight increase in macrophages (F480^+^ CD11b^+^) was shown after exposure to conventional LS, but this rise was more pronounced in the BLS group (*P = 0.04 compared to control). Therefore, exposure to BLS clearly caused profound changes compared to LS primarily in terms of peritoneal inflammatory macrophages recruitment, and IL-17 expressing CD4 cells.

**Table 1:** Quantification of total cell number (×10^6^) composition of cells (%) in mouse peritoneal effluents after 8 weeks of exposure

Cell populations	Groups (Mean ± SD)		
	C	LS	BLS
Total cells (×10^6^)	2.93 ± 1.31	4.36 ± 2.06	5.38 ± 2.56
CD4^+^ (%)	29 ± 1.14	22.13 ± 13.73	17 ± 13.87
CD8^+^ (%)	14.35 ± 3.23	3.45 ± 3.97	6.51 ± 7.37
CD4^+^ IL-17^+^ (%)	1.98 ± 2.46	13.19 ± 8.23*	1.47 ± 2.49
Monocytes (%)	7.45 ± 8.7	10.88 ± 10.09	14.8 ± 11.38
Macrophages (%)	2.03 ± 0.69	8.16 ± 6.84	25.07 ± 16.78*
B cells (%)	37.85 ± 19.45	37.04 ± 23.18	35.03 ± 13.31
	*P < 0.05		

Data show Means ± SD (*n* = 4). Differences were considered statistically significant for *P* < 0.05 using one-way ANOVA. *P < 0.05. (Statistically significant differences were found in LS vs. C and LS vs. BLS for CD4^+^ IL-17^+^, and BLS vs. C in Macrophages)

*C* control, *LS* lactate PD fluid, *BLS* bicarbonate/lactate PD fluid

### Cytokine production changes in BLS-treated mouse

**Fig. 3:**
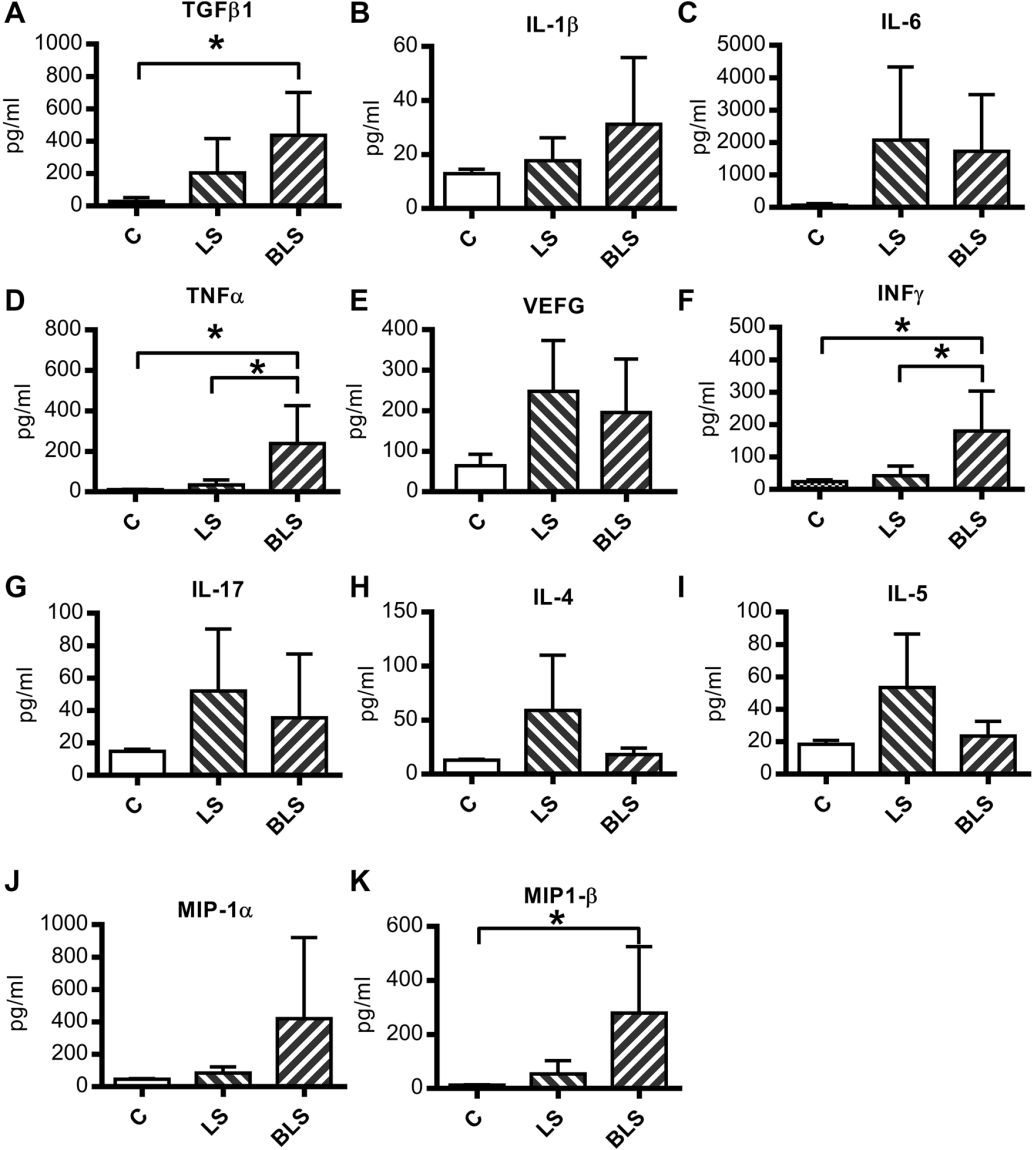
Exposure to a bicarbonate/lactate-buffered solution is associated with increase of pro-inflammatory cytokines. Protein levels of the main pro-inflammatory cytokines detected in effluents collected from mouse after 8 weeks of PD fluid exposure. Cytokines levels (ρg/ml) are represented as means ± SD (*n* ≥ 7) as follows: **a** TGFβ1, **b** IL-1β, **c** IL-6, **d** TNFα, **e** VEGF, **f** INFγ, **g** IL-17, **h** IL-4, **i** IL-5, **j** MIP-1α, **k** MIP-1β. *C* control, *LS* lactate PD fluid, *BLS* bicarbonate/lactate PD fluid. Differences were considered statistically significant for *P* < 0.05 using one-way ANOVA. *P < 0.05

We further explored the differences between the PD fluids in terms of inflammatory cytokine responses. As shown in Fig. 3a, an increment in TGFβ1 levels was detected upon both BLS (435.9 ± 266.3 pg/ml) and LS (204.1 ± 211.8 pg/ml) treatment when compared to control group (C:28.57 ± 22.85 pg/ml) although this was statistically significant for BLS only (*P* = 0.04). A similar pattern was observed for IL-1β that was slightly enhanced in the LS group and further increased upon BLS exposure (Fig. 3b), albeit non-significant. Alternatively, IL-6 and VEGF release increased as a consequence of PD fluid exposure but it did not differ statistically between the PD regimens and control (Fig. 3c, e). On the other hand, a statistically significant increase of TNFα was shown only after BLS exposure but not after LS treatment (C: 10.38 ± 1.25, LS: 33.57 ± 24.82, BLS: 239 ± 186.8 pg/ml; *P* = 0.02) (Fig. 3d).

Emerging evidence points to IL-17 as an important factor in mediating peritoneal inflammation [11]. In this study, the increment found in the CD4^+^ IL-17^+^ cell population upon LS exposure was accompanied by a nominal, but statistically insignificant, increase of the levels of IL-17 measured in the peritoneal effluents (Fig. 3g). We found upregulation of IL-5 in the LS group which was not shown upon treatment with BLS (C: 18.44 ± 2.40, LS: 53.4 ± 33.09, BLS: 23.44 ± 9.13) (Fig. 3i). A similar trend was found for IL-4 (C: 13 ± 0.70, LS: 59.07 ± 51.03, BLS: 18 ± 6.06) (Fig. 3h), indicating that involvement of T helper 2 (Th2) cells was more pronounced upon LS treatment over BLS. On the other hand, the statistically significant high levels of INFγ (Fig. 3f) detected only after BLS exposure suggested that in BLS regimens an important role may be played by T helper 1 (Th1) cells (C: 23.69 ± 5.65, LS: 42.18 ± 30.2, BLS: 180.3 ± 123.5; *P* = 0.01).

The presence of enhanced macrophage population in BLS-treated mouse was further substantiated by the rise of MIP-1α and MIP-1β (also known as CCL3 and CCL4) following BLS treatment although significant changes were only found in the MIP-1β measurements (MIP-1β C: 12.81 ± 1.43, LS: 53.43 ± 49.73, BLS: 278.8 ± 246.8; *P* = 0.04) (Fig. 3j, k). Taken together, between the two PDFs, there are outspoken differences in levels of TGFβ1, TNFα, INFγ, and MIP-1β suggesting that macrophages played a crucial role over other cell types in modulating the response of BLS on peritoneal fibrosis and angiogenesis.

### BLS exposure induced the recruitment of pro-inflammatory macrophages in parietal peritoneum

**Fig. 4:**
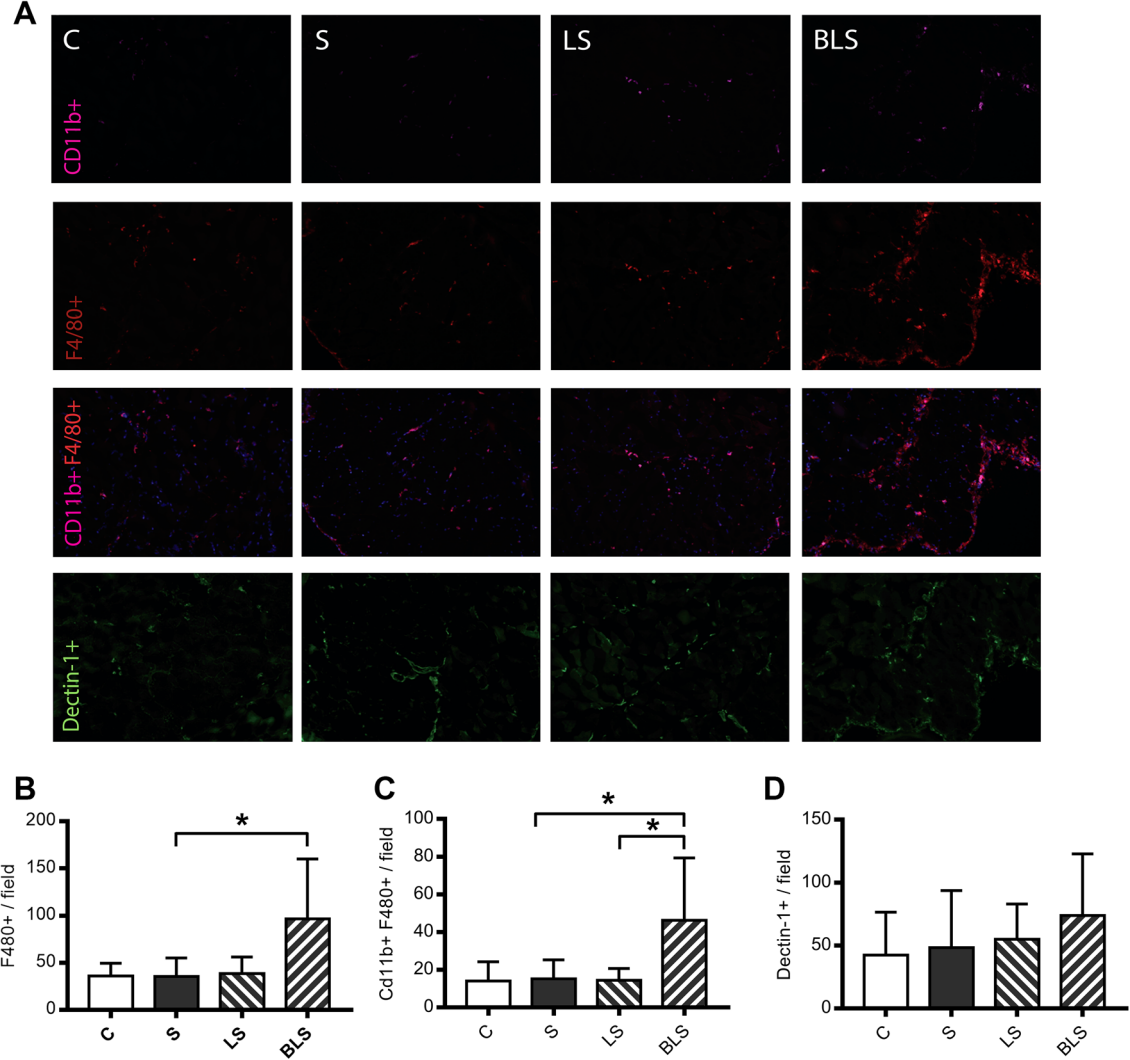
Bicarbonate/lactate-buffered solutions mediate pro-inflammatory macrophages recruitment. Panel **a** shows representative peritoneal membrane macrophages recruitment for each group in the uremic PD mouse model. Peritoneal staining for CD11b (violet), F480 (red), CD11b plus F480 double positive and Dectin-1 (green) are represented in the rows from the top to the bottom panel, respectively. Nuclei were stained with DAPI (blue). Column bars represent number of macrophages measured per field in three different pictures taken per mouse for each group. Significant increase of CD11b and F480 double positive and single F480-positive cells in the BLS group are represented, respectively, in graphs (**b**) and (**c**). Graph **d** shows Dectin-1-positive cells in each group (*C* control, *LS* lactate PD fluid, *BLS* bicarbonate/lactate PD fluid). Magnification ×20. Data show means ± SD (*n* = 10). Differences were considered statistically significant for *P* < 0.05 using one-way ANOVA. *P < 0.05

Given the parallels between the high percentage of macrophages in effluent and the elevated levels of chemokine MIP-1α and 1β, we further explored macrophage subset population in the peritoneum by immunohistochemistry (Fig. 4a). Quantification shows high F480 expression, characteristic of classically activated macrophages (M1) [16], in the peritoneum of BLS-treated mouse (Fig. 4b). In addition, macrophages with classical morphology display high levels of both F480 and CD11b [17], which were significantly increased in the BLS group compared to both the control and the LS groups (Fig. 4c). On the other hand, staining for Dectin-1, a marker for anti-inflammatory (M2) macrophages, did not reveal any significant differences among groups (Fig. 4d). Overall, our results suggest that pro-inflammatory M1 phenotype modulated the response in the peritoneum induced by BLS exposure.

## Discussion

The current study is the first one using a mouse PD exposure model comparing lactate and bicarbonate/lactate-buffered solutions in a uremic setting, which more closely mimics the clinic status of PD patients when compared to the non-uremic animal models. Our findings support the notion that bicarbonate/lactate-buffered solution better preserves the peritoneal integrity status when compared with the conventional PD fluid. Specifically, we demonstrated that the enhanced CD4^+^ IL-17^+^ cell population in effluent detected in LS-exposed mouse was prevented in the BLS group although with no significant changes in IL-17 effluent concentrations. Importantly, increased macrophage population together with high levels of chemokines that regulate migration and infiltration of monocytes/macrophages suggested that macrophages played a key role during BLS treatment. While pro-fibrotic M2 macrophages are thought to promote fibrosis and angiogenesis upon conventional PDF fluid treatment, in this study it was found that in BLS-exposed mouse the pro-inflammatory (M1) phenotype was dominant in the peritoneal tissue over M2 subset suggesting a difference in inflammatory response between different PD fluid exposures.

The accumulation of extracellular matrix and fibrosis is a characteristic peritoneal alteration induced by PD fluid exposure which leads to a progressive remodeling of the peritoneal membrane [1]. As previously demonstrated [15], and confirmed by the present study, daily exposure of conventional PD fluid contributes to the fibrotic response by an accumulation of αSMA-positive cells in the parietal peritoneum. Consistently, an increment of the extracellular matrix protein collagen I in peritoneal tissues of LS-treated mouse was observed. In contrast, daily exposure to BLS fluid prevented the accumulation of both αSMA and collagen I. This is in accordance with previous studies suggesting that the use of low GDPs solutions was associated with less αSMA expression in vitro and less development of fibrotic response in the peritoneal membrane in rats [4, 18, 19]. Importantly, other factors such as the neutral pH present in the more biocompatible fluid may also contribute to the differences observed. In addition, as we reported previously in a rat model with PD [4, 20], daily exposure to BLS prevented new vessel formation, which did occur when LS was used, indicating that BLS regimen is more biocompatible in terms of peritoneal angiogenesis. Our findings suggest reduced angiogenesis and peritoneal fibrosis as markers of better preservation of the structure of the peritoneum after exposure of BLS fluid. Despite the differences reported, no changes in peritoneal thickness were detected among the PD-treated groups. Numerous studies have suggested a relationship between peritoneal structural changes and the increment of fibrosis [21–23]. However, other factors such as the amount of cell infiltrate may contribute to the increment of peritoneal thickness [24]. In addition, daily instillation with normal saline also induces a slight increment of the thickness, indicating that peritoneal remodeling is not exclusive of PD fluid effects, and these additional effects may have masked or overwhelmed any difference between types of PD solution used. Furthermore, we previously reported that uremia per se also contributes to this event [14]. Overall, all those factors may influence the lack of differences in thickness between PD groups.

In vitro experimental research has demonstrated the capacity of neutral pH, bicarbonate/lactate-buffered solutions to maintain mesothelial cellular integrity and function when compared to conventional fluid [18, 25]. Although PD fluid may exert a direct impact on the mesothelial cell stability, evidence points to the inflammatory response as key factor inducing PD-associated pathology. In this regard, in our previous studies, the chronic exposure to standard dialysis fluids resulted in peritoneal Th17 response including elevated IL-17 protein production [11]. Importantly, the modulation of IL-17 during treatment with PD fluid was shown to be an effective therapy for PD-mediated peritoneal fibrosis and angiogenesis [11, 15, 26]. In our study, the increase in CD4^+^ IL-17^+^ cell population observed upon LS treatment was prevented in the BLS groups. Although no significant changes in IL-17 levels were observed between PD fluid exposed animals, it is possible that in this study statistical significance for IL-17 was missed due to too low power.

Alternatively, a striking enhanced INFγ concentration in the effluents of BLS group compared to the LS was found. In parallel, BLS slightly prevented the increment of IL-5 and IL-4 levels detected in the LS group. These findings suggested that inflammatory mechanisms occurring during exposure to bicarbonate/lactate buffer involve Th1 rather than Th2 cell subset while the opposite happened upon exposure to high GDPs lactate solutions. Possibly, this particular inflammatory milieu can explain the differences observed in peritoneal remodeling between PD fluids. In this regard, T helper-related cells interact with many immune cells including macrophages, which also play a crucial role in chronic inflammation-induced fibrosis [27]. Enhanced levels of Th2 cell subsets together with pro-fibrotic cytokine microenvironment contribute to the polarization of peritoneal macrophages towards the anti-inflammatory or pro-fibrotic M2 macrophages subset [27]. Particularly, M2 macrophages subset demonstrated to be dominant in the cellular infiltrate in PD patients and are suggested to drive peritoneal fibrosis [12, 28]. In this study, BLS exposure mediated the increment of the proteins of the chemotactic factors for macrophages MIP-1α and MIP-1β in the peritoneal effluents. It has been shown that in the inflammatory phase, newly attracted macrophages present a more pro-inflammatory (M1) phenotype but only after the switch to M2 they become pro-fibrotic [16, 27, 28]. Interestingly in our experiment, the increase in the percentage of macrophages cell population in the peritoneal effluents after BLS exposure was associated with enhanced recruitment of pro-inflammatory M1 macrophages in the peritoneal membrane. Our results showed indeed an accumulation of macrophages in the parietal peritoneum and prevalence of the pro-inflammatory (F480^+^) over the anti-inflammatory subset (Dectin-1^+^) in the BLS group. In line with these findings, inflammatory M1 macrophages secrete TNFα which were significantly enhanced in the effluent of the BLS-exposed mice when compared to the group undergoing standard PD treatment. Furthermore, M1-polarization, or classical activation, is induced by INFγ which also was significantly incremented upon BLS fluid. In contrast, BLS-treated mouse did have high levels of the pro-fibrotic cytokine TGFβ1, also known to be a stimulus of M2 polarization [27], which, however, as outlined, did not occur. Nevertheless, in some PD studies, increased levels of TNFα, TGFβ1, and INFγ have been interpreted as a consequence of improved mesothelial and macrophages cell function as part of a pro-inflammatory process [5, 29, 30]. However, further studies are needed to confirm the effects of these cytokines during longer periods of PD treatment with a more biocompatible fluid. Overall, these findings suggest that the differences observed between the two PD fluids might rather indicate that an inflammatory process led by an influx of M1-macrophages mainly occurs in the BLS subgroup, while a more fibrotic response takes place in the LS group. Importantly, we previously showed in vitro that only M2 macrophages, and not M1, secrete factors inducing αSMA expression and fibrosis [31]. So, based on these findings, our present results showing a higher prevalence of peritoneal M1 over M2 macrophages in the BLS-treated animals can be interpreted as preventive from the development of fibrosis by BLS exposure as compared to conventional PD fluid.

Our study bears the limitation that the exact mechanism involved in the distinct effects of PD fluid exposure is not defined. Moreover, we did not explore other inflammatory mediators such as T regulatory cells which are important to regulate the activated T-cell expansion [32]. However, we provide a characterization of the key immunological cells dominating the peritoneal response upon standard high GDPs lactate solution and we compare it with a low-GDP bicarbonate/lactate-buffered fluid. Although previous experimental research already suggested the capacity of bicarbonate/lactate-buffered solution in preserving morphologic parameters when compared with the conventional fluid, here we support previous findings and extend the data by using our well-established uremic model combined with long-term exposure to PD fluid [14]. As an additional limitation, no differences in the ultrafiltration capacity were detected between groups. This weakness, however, was previously reported in our previous study in uremic mice and rats with PD [15]. Furthermore, an assumed reduction of the harmful effects of the new generation of PD fluids may explain limited peritoneal thickening after exposure to any PD treatment when compared to relevant effect showed in our previous studies [33, 34]. This event suggests that longer exposure with PD fluid is necessary to fully explore the different effects in thickness and ultrafiltration. Finally, our model would be closer to the clinical situation by the addition of the drainage of the PD fluid. This feature, however, was not included since rodent models of PD absorb the fluid before 24-h post-instillation.

In conclusion, a large difference exists in inflammatory response between conventional and low-GDP bicarbonate/lactate-based PD fluids. The use of the latter solution in our uremic mouse model leads to better preservation of the peritoneal membrane in terms of fibrosis and vascularization. Moreover, peritoneal recruitment of M1 macrophages, higher levels of macrophage-related pro-inflammatory cytokines and lower number of CD4^+^ IL-17^+^ cells might explain the response observed in the peritoneal membrane of BLS-exposed mice. Finally, we provide a better understanding of the inflammatory mediators during the exposure of bicarbonate/lactate low GDPs buffered solution which might help to design new therapeutic approaches favoring the PD treatment.
